# Chronic marijuana usage by human pancreas donors is associated with impaired islet function

**DOI:** 10.1371/journal.pone.0258434

**Published:** 2021-10-27

**Authors:** Meirigeng Qi, John S. Kaddis, Kuan-Tsen Chen, Jeffrey Rawson, Keiko Omori, Zhen Bouman Chen, Sangeeta Dhawan, Jeffrey S. Isenberg, Fouad Kandeel, Bart O. Roep, Ismail H. Al-Abdullah

**Affiliations:** 1 Department of Translational Research and Cellular Therapeutics, Beckman Research Institute, City of Hope, Duarte, California, United States of America; 2 Department of Diabetes Immunology, Beckman Research Institute, City of Hope, Duarte, California, United States of America; 3 Department of Diabetes and Cancer Discovery Science, Beckman Research Institute, City of Hope, Duarte, California, United States of America; 4 Department of Diabetes Complications & Metabolism, Arthur Riggs Diabetes & Metabolism Research Institute, Beckman Research Institute, City of Hope, Duarte, California, United States of America; Centre de Recherche des Cordeliers, FRANCE

## Abstract

We investigated the effect of chronic marijuana use, defined as 4 times weekly for more than 3 years, on human pancreatic islets. Pancreata from deceased donors who chronically used marijuana were compared to those from age, sex and ethnicity matched non-users. The islets from marijuana-users displayed reduced insulin secretion as compared to islets from non-users upon stimulation with high glucose (AUC, 3.41 ± 0.62 versus 5.14 ±0.47, p<0.05) and high glucose plus KCl (AUC, 4.48 ± 0.41 versus 7.69 ± 0.58, p<0.001). When human islets from chronic marijuana-users were transplanted into diabetic mice, the mean reversal rate of diabetes was 35% versus 77% in animals receiving islets from non-users (p<0.01). Immunofluorescent staining for cannabinoid receptor type 1 (CB1R) was shown to be colocalized with insulin and enhanced significantly in beta cells from marijuana-users vs. non-users (CB1R intensity/islet area, 14.95 ± 2.71 vs. 3.23 ± 0.87, p<0.001). In contrast, CB1R expression was not co-localized with glucagon or somatostatin. Furthermore, isolated islets from chronic marijuana-users appeared hypertrophic. In conclusion, excessive marijuana use affects islet endocrine phenotype and function *in vitro* and *in vivo*. Given the increasing use of marijuana, our results underline the importance of including lifestyle when evaluating human islets for transplantation or research.

## Introduction

The marijuana plant *Cannabis sativa* contains over 100 components termed cannabinoids [1]. Cannabinoids interact with cannabinoid receptors, including type 1 (CB1R) and type 2 (CB2R), on the cell membrane to regulate several signaling pathways [2] that modulate food intake, increase appetite [3], and regulate lipid and glucose homoeostasis and insulin sensitivity [4]. Medical and recreational marijuana use has increased significantly, especially upon its legalization [5]. This has corresponded with increased interest in understanding the possible effects of chronic marijuana exposure, particularly in relation to organ donation [6].

Access to human tissue samples and the development of islet and pancreas distribution networks increased our understanding of human islet biology [7, 8]. To minimize spurious results, standardized reporting criteria of human islet characteristics were promulgated [9, 10]. The criteria included the age, gender, cause of death, hospitalization, medical treatment of the organ donor and graft procurement details. The criteria do not take into account donor lifestyle, especially drug usage. This is not a trivial concern. Numerous publications that had impact on clinical practice utilized pancreata from donors with type 1 diabetes [11], up to one third of whom were drug users [12]. The impact of endocannabinoid signaling on pancreatic islets remains controversial with data suggesting beneficial and adverse effects [13–20]. Yet, few studies have investigated the effect of heavy marijuana use on the functionality of donated organs and especially isolated pancreatic islets transplanted into diabetic recipients.

Herein, we tested the hypothesis that chronic marijuana use, defined as 4 times weekly for more than 3 years, among pancreas organ donors adversely altered islet characteristics and function.

## Materials and methods

### Donor selection

The islet isolation outcomes of 275 brain-dead deceased donor pancreases received between 2006 and 2016 at City of Hope National Medical Center were analyzed. Informed consent for the use of human tissue was obtained from the donor next of kin and ethics approval for this study was granted by the Institutional Review Board of City of Hope (IRB # 01046). Donors with elevated HbA1c (>6.5%) (n = 11), elevated serum sodium levels (>160 mEq/L) (n = 15), and donation after cardiac death or unknown down-time criteria (n = 16) and infrequent marijuana usage (n = 27) were excluded from this analysis. Usage was considered infrequent if the donor consumed marijuana 5–10 times per year for less than 3 years. The remaining 206 donors were divided into two groups based on the medical and social histories documented in the donor charts provided by the organ procurement organizations at the time of placing the pancreas offer. Donors with extensive marijuana use (4 times weekly for more than 3 years) were defined as the chronic marijuana-user group (n = 26), while donors with no documented history of marijuana use were defined as the non-user group (n = 180). Matched subsets of donors were then identified in the 2 groups to limit bias effect estimates from the covariates of age, sex, and ethnicity. Given the conflicting reports on the relationship to marijuana use, BMI was not retained as a covariate used in the matching process [21, 22]. Propensity score [23], defined as the conditional probability of being a non-marijuana user given the three covariates, was calculated for all donors. The logit of the propensity score was used to match donors across the two groups in a 2:1 ratio using a randomly sorted nearest-available-neighbor matching method without replacement. A caliper width equal to 0.20 of the standard deviation of the logit value was shown to be ideal in a 1:1 matching scenario [24]. Here, two times the caliper width from 1:1 was used. Freely available SAS macros were employed for this analysis [25]. These criteria identified 19 non-users and 10 chronic marijuana-users (29 in total, 2:1 matching in all but 1 marijuana donor), all non-diabetic, for further study.

### Islet isolation, characteristics, and function

Islet isolation and purification were performed according to standard protocols [26, 27]. Islets were cultured in Connaught Medical Research Laboratories (CMRL)-1066 supplemented media for 24–48 hours prior to assessment. Islet purity was determined by estimating the proportion of dithizone (DTZ)-containing (red-stained) islets to all tissues present (islets, acinar, and ductal cells) as described [28, 29]. In each isolation, duplicate islet samples were taken to determine islet size as described [29]. Islet viability was determined using fluorescein diacetate (FDA) and propidium iodide (PI) (Sigma-Aldrich, St Louis, MO, USA) staining as described [30]. Briefly, 100–200 IEQ islets were stained with FDA/PI fluorescent dye solution for 5 minutes at 22°C and then washed with PBS. The percent viability of an islet sample was calculated as follows: viability (%) = 100 –([PI-positive area/islet area] × 100). FDA-positive (live cells) or PI-positive areas (dead cells) were calculated using imaging software (cellSens, Olympus) [31]. Islet perifusion studies to measure dynamic insulin secretion after high glucose (16.8 mM) and KCl (25 mM) stimulation were performed as described [32]. Briefly, 200 IEQ islets were loaded into a column with Bio-Gel P4 beads (Bio-Rad, Cat# 150–4124). The islets were perifused for 1 hour with low glucose (3mM) using Krebs-Ringer’s Buffer Solution (pH 7.4) at 37°C. The column was washed initially with 3mM glucose for 10 minutes, followed by 16.8mM glucose for 20 minutes and then by 3mM glucose for 25 minutes before switching to 25mM KCl plus 16.8mM glucose. Elutions were collected every minute and the concentration of insulin in each sample was measured using an ELISA kit (Mercodia Inc., Winston Salem, NC, USA, Cat# 10-1113-10).

### *In vivo* islet function

To assess islet function, transplantation of isolated human islets into male nonobese diabetic/severe combined immunodeficient diabetic (NOD SCID) (NOD CB17-*Prkdc^scid^*) mice (8–10 weeks old) was performed. All mice were purchased from Jackson lab (Jackson Laboratory, Bar Harbor, ME) and housed under specific pathogen-free conditions at the Animal Resources Center of the Beckman Research Institute of City of Hope. Animal experiments were carried out with the approval of the Institutional Animal Care and Use Committee of City of Hope (IACUC # 01020). Diabetes was induced by intraperitoneal injection of 50 mg/kg streptozotocin (STZ; Sigma-Aldrich, St Louis, MO) for three consecutive days. Mice with hyperglycemia (blood glucose > 350 mg/dL) on two consecutive days were transplanted with 1,200 IEQ of single donor human islets placed under the left kidney capsule [33]. Islets from each donor pancreas were transplanted into at least 2 mice. Following islet transplantation, blood glucose was determined 2–3 times per week for 28 days using a glucometer (LifeScan, Inc., Milpitas, CA, USA). Animals that maintained blood glucose levels <200 mg/dL were labeled as diabetes free if at least 2 consecutive readings were obtained [33]. Failure to reverse diabetes was defined as blood glucose levels in mice that a) never dropped below 200 mg/dL, or b) dropped below 200 mg/dL for 2 consecutive readings but then later rose above this value (relapse). For each batch of islets isolated from a single donor, the ratio of the number of mice that reversed diabetes divided by the total number of mice transplanted was calculated [33]. Hyperglycemia was confirmed in all the mice that reversed diabetes following removal of the islet graft bearing kidney. A total of 30 and 50 mice received human islets from 10 marijuana-users and 19 non-users respectively. The animals were anesthetized with 2% isoflurane (Patterson Veterinary, Greeley, CO) inhalation and pain was alleviated by injecting 0.5 mg/kg of buprenorphine (ZooPharm, Laramie, WY) subcutaneously. All the animals were sacrificed by an overdose of isoflurane.

### Immunofluorescent staining

Human pancreatic tissues were fixed in 10% formalin prior to preparation of paraffin blocks and tissue sectioning. Tissue sections (5 μm) were deparaffinized with Histo-Clear (National Diagnostic, Atlanta, GA) and rehydrated. Super-sensitive wash buffer (1×) (Biogenex, Fremont, CA, Cat # HK 583-5K) was used to wash the tissue slides. Antigen retrieval was performed for 40 minutes with citric acid-based antigen unmasking solution (Vector Lab, H3300, pH 6.0) using a steamer (Black & Decker, Towson, MD). Sections were treated with CAS-Block Histochemical Reagent (Invitrogen, Cat# 00–8120) for 20 minutes at room temperature to reduce background signal. For pancreatic tissue (six chronic marijuana-users and five non-users), sections were incubated over night at 4°C with primary antibodies including rabbit anti-CB1R, which targets the human extracellular N-terminus of CB1R (1:200 dilution, Alomone Labs, Israel, Cat# ACR-001), guinea pig anti-insulin (1:400 dilution, Abcam, Cat # ab195956), mouse anti-glucagon (1:2,000 dilution, Sigma), rat anti-somatostatin (1:50 dilution, BioRad). The following secondary antibodies were used for human pancreatic tissue: Cy3-conjugated AffiniPure donkey anti-rabbit IgG (H+L) antibody (1:200 dilution, Jackson ImmunoResearch Lab, Code # 711-165-152), ALEXA 488-conjugated goat anti-rabbit (1:200 dilution, Invitrogen), ALEXA 488-conjugated donkey anti-guinea pig (1:200 dilution, Jackson Lab), ALEXA 488-conjugated donkey anti-mouse antibody (1:200 dilution, Jackson Lab), and ALEXA 555 goat anti-rat antibody (1:200 dilution, Invitrogen). Primary and secondary antibodies were diluted in CAS-Block Histochemical Reagent. Fluoroshield containing DAPI (Sigma Aldrich, St. Louis, MO, Cat # F6057) was used to stain nuclei and for coverslip mounting. To validate the CBR1 antibody, staining of human brain cortex that is known to express CB1R (positive control) and isotype control was also performed. Tissue sections were examined under a Zeiss LSM 700 Confocal Microscope (Carl Zeiss). Multiple images were taken with the objective lens set at 10× and 20× magnification.

### Quantitative analysis of confocal images

Analysis of immunofluorescent-stained tissue sections was performed using ImageJ software [34]. Briefly, to determine the expression of CB1R and insulin, corresponding confocal images were opened using the ImageJ application. On each image containing human pancreatic tissue, the islets (pancreatic tissue) were selected using the polygon selection tool. After selecting “RGB Measure” from the Plugins/Analyze menu, the values of fluorescent intensity according to different fluorescent channels and area of interest were obtained. CB1R expression was determined in islets from each donor organ tissue section and expressed as CB1R intensity/islet area. Sections from five non-user and six marijuana-user samples were counted and analyzed. For each group (marijuana-users or non-users), 5 separate tissue sections were analyzed. In each section, at least 2 islets were counted for final analysis. The CB1R intensity per islet area was compared between two donor groups.

### Statistical methods

Categorical variables were described using number and percent, and continuous factors summarized using the mean (± standard deviation) for normally distributed or median (minimum, maximum) for skewed distributions. Data in the figures were expressed as mean ± SEM. Statistical testing was performed for continuous variables using a two-sample t test with a pooled or Satterthwaite corrected p-value if parametric, or the Kruskal-Wallis test if non-parametric. Categorical variables were analyzed using a Pearson’s chi square test if values in all cells exceeded 5, or by Fisher’s exact test if values in one or more cells was <5. P-values were not calculated if values from 2 or more cells were equal to 0. All reported p-values are two-tailed and significant if <0.05. All analyses were performed using SAS software version 9.4 (SAS Institute, Cary, NC) or GraphPad Prism version 8.0 (GraphPad, La Jolla, CA). All data figures were generated using GraphPad Prism.

## Results

### Chronic marijuana use alters morphology and *in vitro* islet function

**Fig 1:**
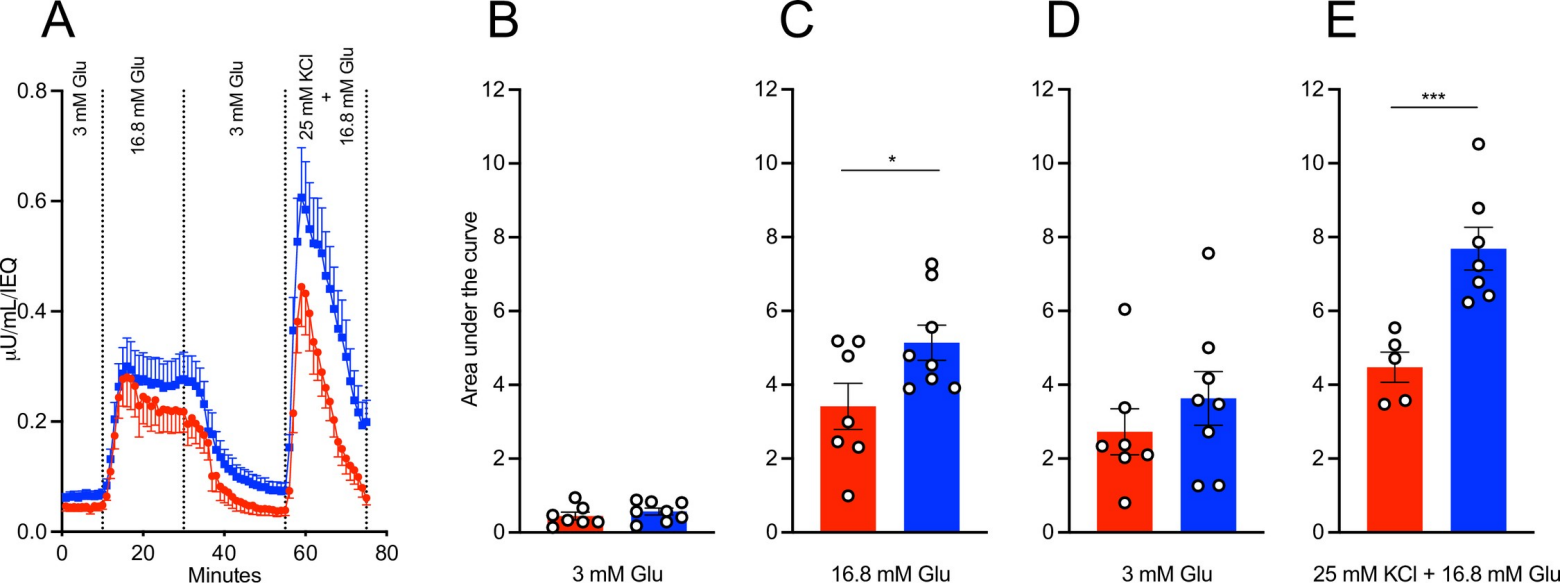
Islets from chronic marijuana-users have lower responses upon *in vitro* challenge with secretagogues. (A) Isolated islets from chronic marijuana-users (red) and non-users (blue). After 48 hours of culture, islets were perifused sequentially with Krebs-Ringer’s Buffer Solution (pH 7.4) containing 3 mM glucose, 16.8 mM glucose, 3 mM glucose, and 25 mM KCl plus 16.8 mM glucose, respectively. Insulin secretion was quantified by ELISA and standardized by islet IEQ number. Area under the curve of secreted insulin on perifusion with (B) 3 mM glucose; (C)16.8 mM glucose; (D) 3 mM glucose; (E) 25 mM KCl plus 16.8 mM glucose in panel A. Marijuana-users (n = 7) and non-users (n = 8). * p<0.05; *** p<0.001. All analyses were performed using GraphPad Prism version 8.0. A p<0.05 was considered significant. The values are expressed as the mean ± SEM.

There were no significant differences in demographic characteristics including BMI, cause of death, HbA1c level, blood glucose at admission, and cold ischemia time between non-users and marijuana-users (Table 1). The pancreas weight and islet count (post-isolation count, post-culture count, and percent recovery) were not significantly different between organs from non-users and marijuana-users (Table 2). Islets from chronic marijuana-users appeared hypertrophic (S1 Fig). The percentage of islets recovered after culture was not significantly different between marijuana-users and non-users (Table 2). The purity and viability of isolated islets were similar between non-user and marijuana-user groups (Table 2). Islets from marijuana-users showed decreased insulin secretion following 16.8 mM glucose stimulation (AUC, 3.41 ± 0.62 versus 5.14 ± 0.47) (Fig 1A and 1C, p<0.05). Furthermore, chronic marijuana-user islets showed significantly reduced insulin secretion upon KCl and 16.8 mM glucose stimulation compared to non-users (AUC, 4.48 ± 0.41 versus 7.69 ± 0.58) (Fig 1E, p<0.001).

**Table 1:** Clinical characteristics of organ donors.

Name	(%)*, Mean (± 1 SD)*, or Median (min, max)*				*P*-value
	n	Non-users	n	Marijuana-users	
Age at death (yrs) ^†^	19	40.6 (±11.5)	10	40.1 (±12.5)	0.909
Sex ^†^					0.999
Female	6	32%	3	30%	
Male	13	68%	7	70%	
Ethnicity ^†^					0.999
Caucasian	13	69%	6	60%	
Hispanic/Latino	5	26%	3	30%	
African American	1	5%	1	10%	
Body Mass Index (kg/m^2^)	19	30.4 (±5.6)	10	28.6 (±4.9)	0.400
Cause of death					0.218
Cerebrovascular/stroke	5	26%	5	50%	
Anoxia	2	11%	2	20%	
Head Trauma	12	63%	3	30%	
Blood glucose at admission (mg/dL)	19	202 (±60)	10	191 (±91)	0.682
Pancreas function after admission**					
Blood glucose (mg/dL)	19	234 (±53)	10	258 (±62)	0.274
Amylase (U/L)	19	114 (28, 679)	9	134 (47, 298)	0.417
Lipase (U/L)	19	37 (8, 1145)	10	23 (9, 91)	0.155
HbA1c (%) (mmol/mol)	19	5.4 (±0.3)	9	5.3 (±0.5)	0.771
		35.3(±3.6)		34.7(±5.5)	0.711
Other drug use in medical history					NR
None	19	100%	5	50%	
Cocaine	0	0%	1	10%	
Methamphetamines	0	0%	4	40%	
Cardiac arrest					0.352
No	16	84%	6	67%	
Yes	3	16%	3	33%	
Cardiac arrest duration (min)	19	0 (0, 24)	9	0 (0, 20)	0.411
Hospitalization length (days)	19	3.4 (±2.5)	10	3.1 (±1.0)	0.684
Cold ischemia time (hrs)***	19	9.5 (±3.8)	9	7.5 (±3.0)	0.161

* % reported for all categorical variables; use of mean or median based on evaluation of normal distribution.

^†^ Factors used for matching.

**Highest recorded extreme value analyzed.

***Defined as time from aortic cross clamping to end of laboratory organ cleaning.

P-values were not calculated if values from 2 or more cells were equal to 0 and indicated with Not Reported (NR).

**Table 2:** Islet isolation assessment of pancreata from marijuana users and non-users.

Name	(%)*, Mean (± 1 SD)*, or Median (min, max)*				p-value
	n	Non-users	n	Marijuana-users	
Pancreas weight (grams)**	19	102.5 (±24.9)	10	99.3 (±25.7)	0.753
Islet Count					
Post isolation count (total IEQ)	19	268,723 (±113,147)	10	297,454 (±216,133)	0.701
Post culture count (total IEQ)	19	176,546 (±102,430)	10	171,893 (±130,662)	0.917
Percent Recovery***	19	65% (±21.2)	10	58% (±23)	0.301
Quality measurements					
Purity (%)	19	76% (±12)	10	72% (±15)	0.419
Viability (%)	19	95% (±4)	10	95% (±3)	0.757

* % reported for all categorical variables; use of mean or median based on evaluation of normal distribution.

**Weight taken after cleaning of pancreas.

***Defined as post-culture count divided by post-isolation count.

### Islets from chronic marijuana-users are less effective in reversing diabetes in mice

**Fig 2:**
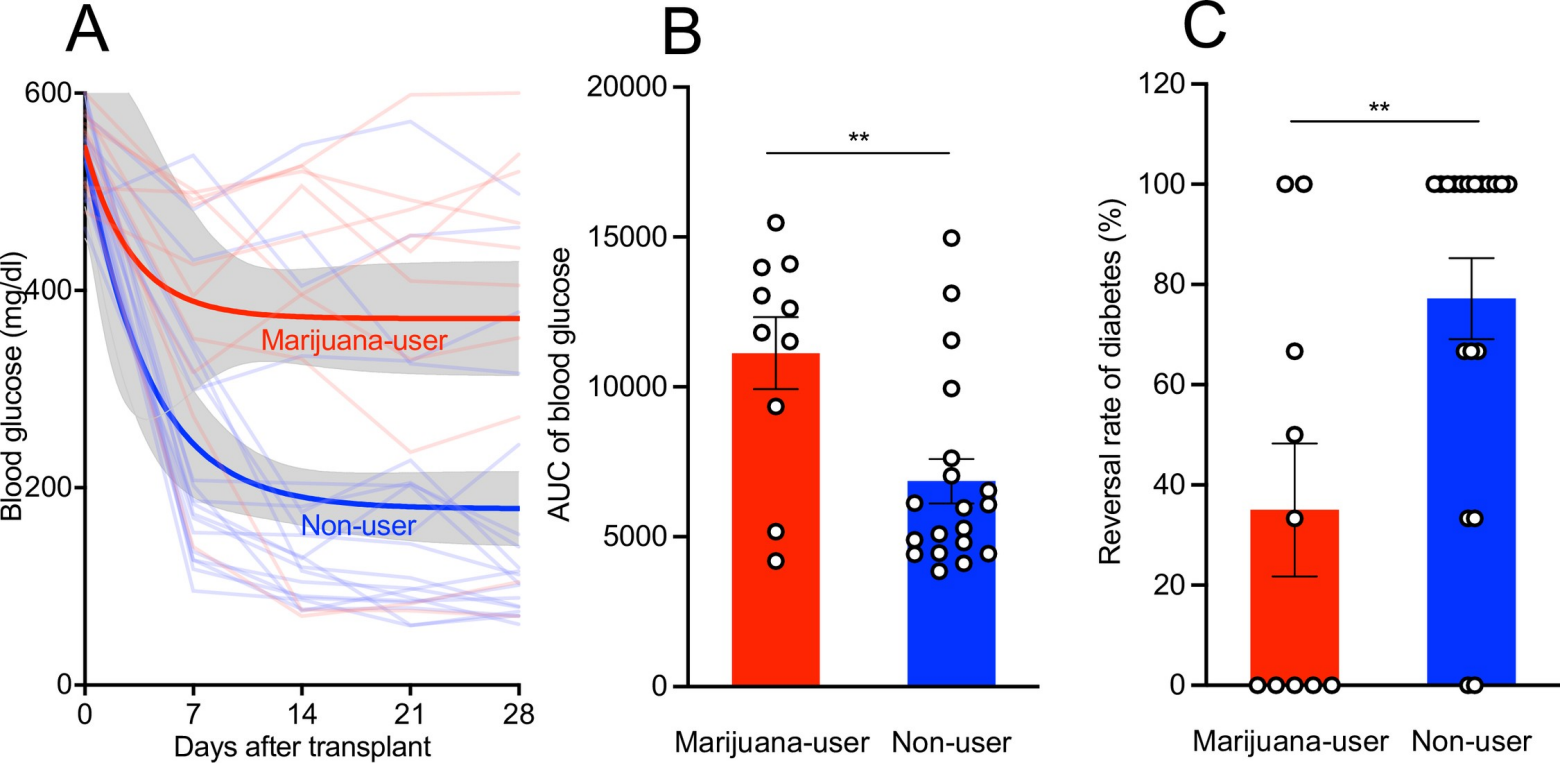
Islets from chronic marijuana-users have diminished graft function following transplantation into diabetic NOD SCID mice. Isolated islets (1,200 IEQ) from non-users and marijuana-users were transplanted beneath the renal capsule of the left kidney of diabetic NOD SCID mice. (A) Blood glucose levels were determined periodically for 28 days post-transplantation. The highlighted areas are the range of standard error of mean of each donor group. (B) Calculated area under the curve of blood glucose levels, marijuana-user (n = 30 mice received human islets from 10 donors); non-user (n = 50 mice received human islets from 19 donors). Each dot in the bar graph represents the average AUC of blood glucose from all the mice transplanted with islets from a single donor. (C) The mean reversal rate of diabetes in the mice after islet transplantation. For each batch of islets isolated from a single donor, the ratio of the number of mice that reversed diabetes divided by the total number of mice transplanted was calculated. Then the mean reversal rates of diabetes were averaged from marijuana-users (10 donors) and non-users (19 donors). Each dot in the bar graph represents the reversal rate of diabetes in single donor. **p<0.01. All analyses were performed using GraphPad Prism version 8.0. A p<0.05 was considered significant. The values were expressed as the mean ± SEM.

To evaluate the islet function *in vivo*, isolated human islets were transplanted into diabetic NOD SCID mice. Animals transplanted with islets from chronic marijuana-users showed higher blood glucose levels for an extended period of time compared to mice that were transplanted with islets from non-users (Fig 2A). This was also reflected by the greater area under the curve (AUC) of the overall blood glucose levels (Fig 2B, p<0.01). The reversal rate of diabetes in mice transplanted with islets from chronic marijuana-users was 35 ± 13%, which was significantly lower than the rate of diabetes reversal in animals receiving islets from non-users (77 ± 8%, p<0.01) (Fig 2C). This is despite that the number of islets transplanted under kidney capsule were the same in both groups (1,200 IEQ).

In the cohort of chronic marijuana users, half the individuals also used cocaine and methamphetamine. Although effects from other drugs is conceivable, further analysis showed that the diabetes reversal rates of two groups following transplantation (use of marijuana only and marijuana plus other drugs) were similar (S2 Fig). This suggests that chronic marijuana use alone has negative effects on human islet function after transplantation.

### Chronic marijuana use is associated with increased CB1R expression in human islet beta cells

**Fig 3:**
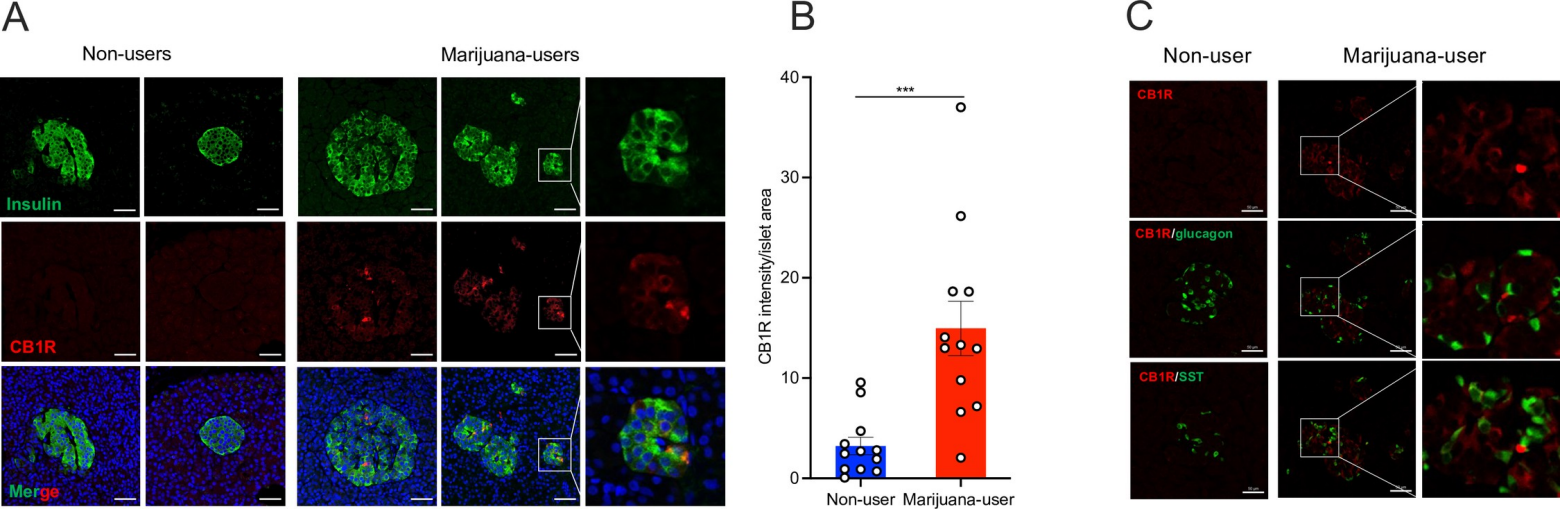
Islets from chronic marijuana-users display increased CB1R expression on islet beta cells. (A) Immunofluorescent staining of human pancreatic tissue from individuals with a history of non-use and chronic marijuana use. The sections were stained for CB1R (red) and insulin (green). Details of the staining are shown in the enlarged images. Two islet images are presented from non-users and marijuana-users. Confocal images were taken using Zeiss LSM 700. Scale bars, 50 μm. (B) Quantitative analysis of CB1R expression. The CB1R intensity per islet area was compared between the two donor groups. Samples from five non-user donors and from six marijuana-user donors were counted for the analysis. *** p<0.001. The values were expressed as mean ± SEM. Each dot in the bar graph represents calculated value from an islet. (C) Immunofluorescent staining of human pancreatic tissue from individuals with a history of chronic marijuana use and non-use. The sections were stained for CB1R (red), glucagon (green), and somatostatin (green). Details of the staining are shown in the enlarged images. Images are presented from samples from four non-users and four marijuana-users. Confocal images were taken using a Zeiss LSM 700 microscope. Scale bars, 50 μm.

Cannabinoid-meditated activation of CB1R in human islets or beta cells remains little studied and controversial [13, 35]. To evaluate the potential effects of chronic marijuana use on CB1R expression in human islets, we performed immunofluorescence staining for CB1R with insulin, glucagon, and somatostatin on pancreatic sections from non-users and marijuana-users. CB1R expression was scarcely detected in islets from non-users. In contrast, islets from chronic marijuana-users showed CB1R expression co-localized exclusively to insulin positive cells (Fig 3A) and significantly increased compared to non-user islets (CR1R intensity/islet area, 14.95 ± 2.71 vs. 3.23 ± 0.87, p<0.001) (Fig 3B). Moreover, immunofluorescent staining of CB1R concurrent with glucagon and somatostatin showed that CB1R co-localization was not found in alpha or delta cells respectively (Fig 3C). CB1R expression was confirmed in human brain cortex tissue (positive control) (S3 Fig) [36].

## Discussion

Cannabinoids are involved in a variety of physiological functions including metabolism [37], behavior [38], emotion, pain [39], motor coordination [40], and insulin sensitivity [41]. However, the effects of chronic marijuana use on human pancreatic tissue and islets have not been investigated. In particular, CB1R expression in human islets was uncharacterized. We report a negative relationship between chronic marijuana use and human islet morphology and function. Islets from chronic marijuana-users, despite normal donor HbA1c levels, stained faintly with DTZ. Also, they appeared hypertrophic and morphologically similar to islets from individuals with T2D [33]. Dynamic insulin secretion studies showed that islets from chronic marijuana-users mounted an inferior insulin response to high glucose and KCl compared to islets from non-users. Consistent with this, increased cannabinoid signaling inhibited glucose response in islet-like cells [17] and promoted beta cell death in murine insulinoma cells [15]. Furthermore, ablation of beta cell CB1R expression enhanced islet function in mice [42]. As well, clinical data suggested that cannabis use was not linked to the risk of developing type 2 diabetes (T2D) [43, 44]. In our study, none of the donors had a history of T2D and their HbA1c levels were all below 6.5%. Regardless, the results of our study extend the field revealing a functional defect when islets from chronic users were challenged with high glucose. Further, when islets from chronic marijuana-users were transplanted into immune deficient diabetic mice glucose imbalance was greater and more persistent. Suggesting a mechanistic link, islets from chronic marijuana-users showed substantially more CB1R expression in insulin positive cells, while overall displaying fewer insulin positive cells compared to islets from non-users. These data indicate enhanced cannabinoid signaling in human islets chronically exposed to marijuana. Further, these data bear strongly upon reports suggesting cannabinoid signaling negatively altered tissue transplantation [45].

Employing IF or cell sorting, CB1R expression was found restricted to beta cells [14, 16, 46–48]. Our histological analysis of human islets found little to no CBR1 expression in islet alpha and delta cells with consistent expression in insulin positive beta cells. These data suggest that CBR1 expression is modified on chronic exposure to marijuana. In this study, the diabetic reversal rate of transplanted islets from users was lower (35%) compared to the rate of transplanted islets from non-users (77%). The mechanism behind this finding remains to be investigated. However, cannabidiol limited angiogenesis by inhibiting endothelial cell growth and migration [49] and cannabis reduced expression of the pro-angiogenetic factor vascular endothelial growth factor [50]. Cannabinoid signaling also limited cancer-related angiogenesis [51] and fetal ovarian vascularity [50]. Therefore, it is conceivable that marijuana negatively alters angiogenetic signaling in islets. This is relevant as transplanted islets require rapid angiogenesis to restore perfusion and function [52]. Advanced age is associated with decreased wound healing and angiogenesis, a process driven, in part, by a loss of self-renewal and upregulation of anti-angiogenic signaling [53]. While not assessed, it would be reasonable to determine if chronic marijuana use perturbs anti-angiogenic signaling in human islets.

Interestingly, we found that islets from marijuana-users were larger than the islets from non-users. This is of likely import, as in the context of graft survival post transplantation, larger islets are recognized as inferior to small islets. This could account, in part, for the lower diabetes reversal rate in mice receiving islets from users [54, 55]. Beyond the negative effects on human islets shown here, other adverse health consequences have been attributed to chronic marijuana use [56]. Marijuana use was associated with reduced sperm count in young man [57]. Exposure to tetrahydrocannabinol or similar marijuana-derived molecules impaired maturation of GABA function in the prefrontal cortex, an important region of the brain involved in complex behaviors and decision making [58]. Survival of liver transplants was increased in recipients that refrained from marijuana use [59] suggesting post-transplantation exposure to marijuana negatively influenced the outcome. Relevance to the present findings, chronic cannabinoid use increased the risk of diabetic ketoacidosis in adults with T1D [60]. In the present study *in vitro* quality and functionality of islets from chronic marijuana users was assessed along standard transplantation release criteria. It is not known if the adverse effects that chronic marijuana exposure had upon human islets is reversable. It is possible that drug abuse induces epigenetic changes [61] which may not revert with abstinence. As well, it will be important to determine if acute marijuana exposure alters human islet function.

In summary, islets from individuals with a history of chronic excessive marijuana use displayed deteriorated islet function concurrent with induction of CB1 receptor on islet beta cells. Given the increasing use of marijuana, our data highlight the importance of considering lifestyle when assessing pancreas islet grafts for transplantation and research.

## Supporting information

S1 Fig Islet size distribution. The chronic marijuana-users presented with significantly more islets in the size range 151–200 μm (p = 0.027) and >400 μm (p = 0.046), and significantly fewer islets in the 50–100 μm size range (p = 0.021). The numbers of independent experiments were 10 and 19 in marijuana-user and non-user groups, respectively.
(PDF)

S2 Fig Islets from donors using marijuana alone and donors using marijuana plus other drugs show similar impairment in restoring glycemic control after transplantation into diabetic NOD SCID mice. Blood glucose levels of non-users (n = 19, blue bold line), marijuana use only (n = 5, red bold line), and marijuana plus other drugs (n = 5, orange bold line). Mean values from all mice per donor are plotted as one line on the graph (n = 29 donor lines total). The bold line represents a locally weighted scatterplot smoothing (LOESS) fit, and the spread around each line the 95% CI.
(PDF)

S3 Fig CB1R expresses in human brain cortex tissue and islets. Immunofluorescent staining of human brain cortex tissue and pancreatic tissue from individuals with a history of chronic marijuana use. The sections were stained for CB1R (red). Isotype control was also used. Nuclei are shown in blue. Confocal images were taken using Zeiss LSM 700. Scale bars, 50 μm.
(PDF)
