# Supplementary figures and images for: Chronic marijuana usage by human pancreas donors is associated with impaired islet function
Source: PLoS One. 2021 Oct 27;16(10):e0258434. doi: 10.1371/journal.pone.0258434 (PMC8550598; doi:10.1371/journal.pone.0258434)

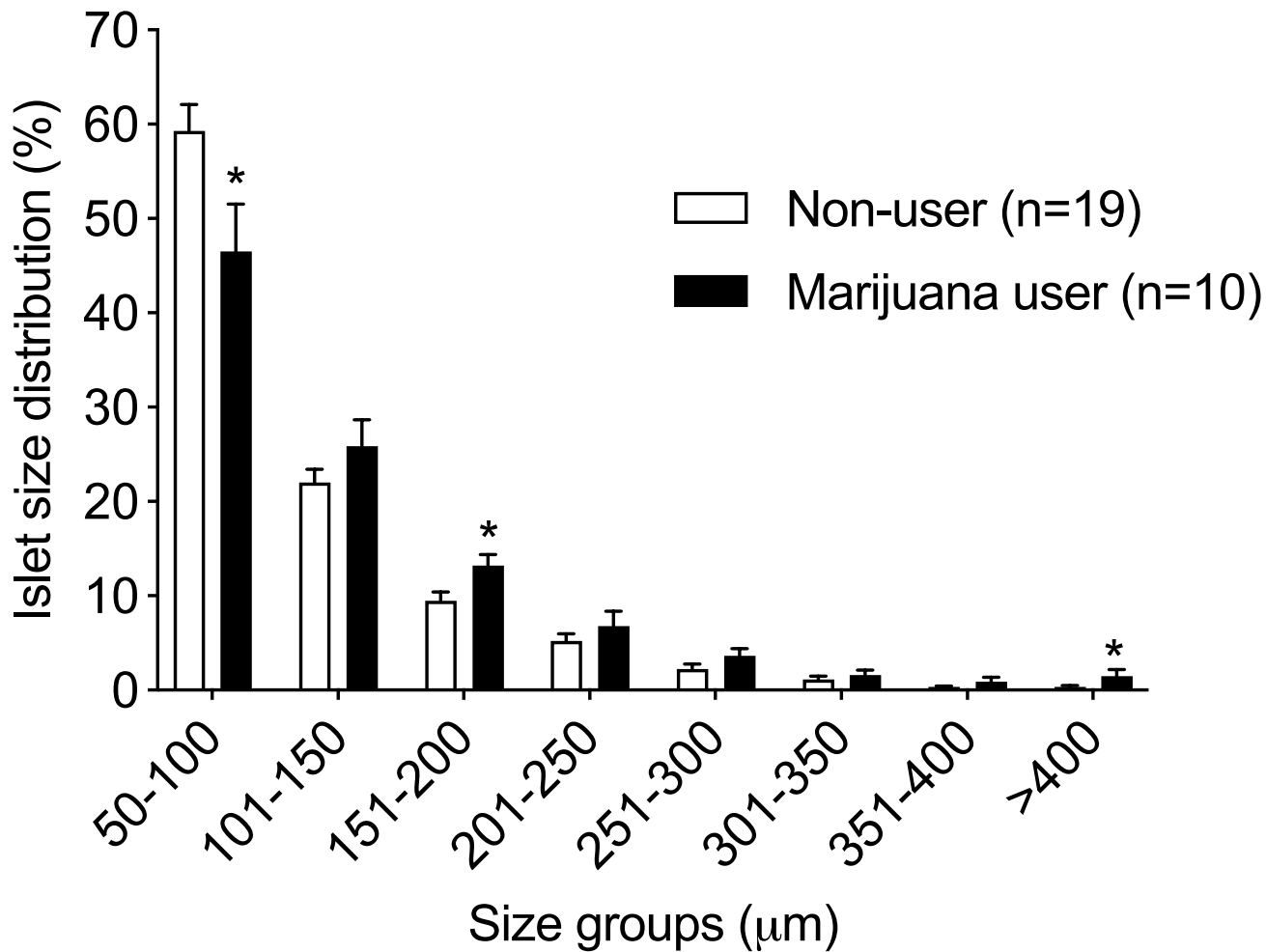

Supplement: S1 Fig — The chronic marijuana-users presented with significantly more islets in the size range 151–200 μm (p = 0.027) and >400 μm (p = 0.046), and significantly fewer islets in the 50–100 μm size range (p = 0.021). The numbers of independent experiments were 10 and 19 in marijuana-user and non-user groups, respectively. (PDF) [file pone.0258434.s001.pdf]

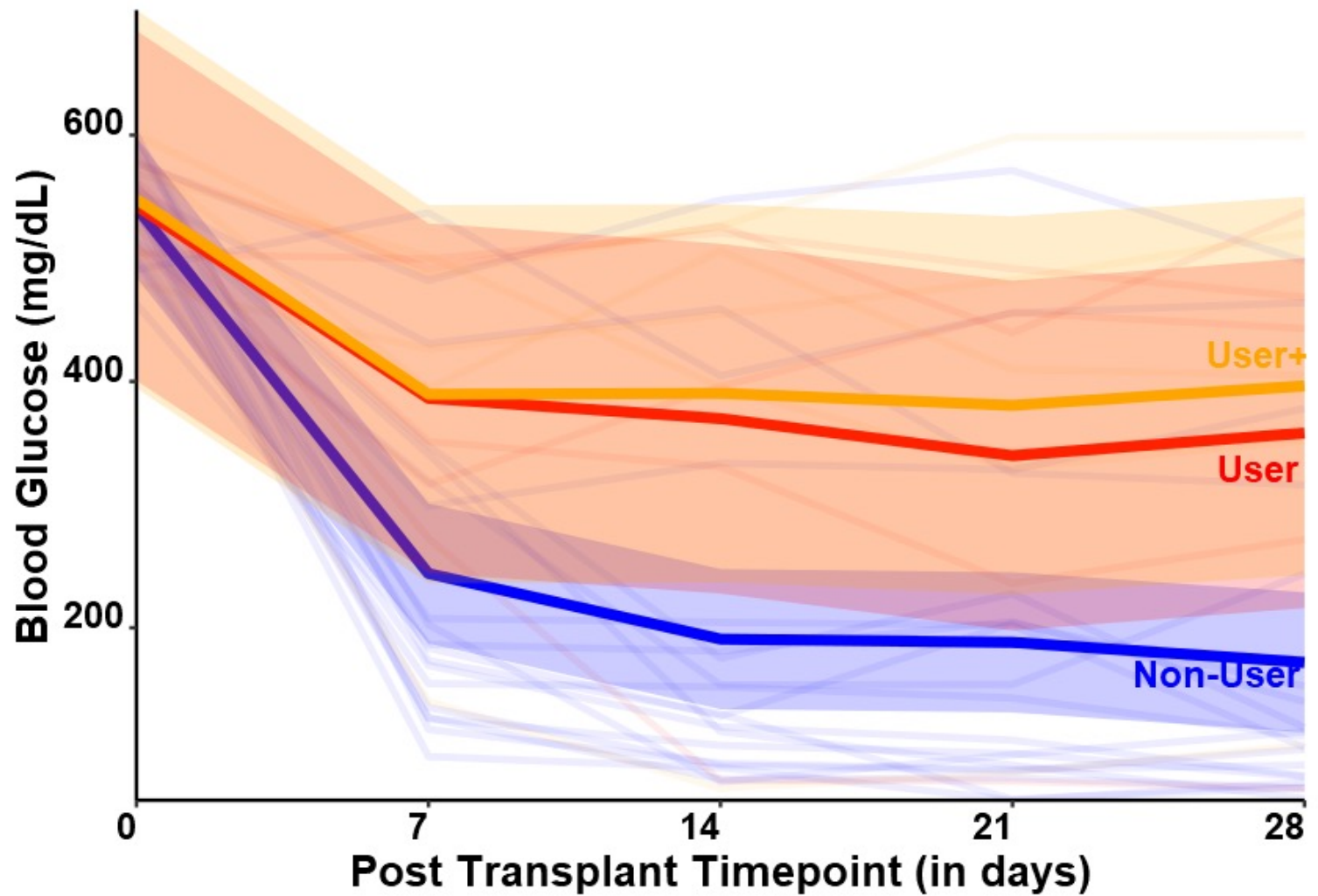

Supplement: S2 Fig — Blood glucose levels of non-users (n = 19, blue bold line), marijuana use only (n = 5, red bold line), and marijuana plus other drugs (n = 5, orange bold line). Mean values from all mice per donor are plotted as one line on the graph (n = 29 donor lines total). The bold line represents a locally weighted scatterplot smoothing (LOESS) fit, and the spread around each line the 95% CI. (PDF) [file pone.0258434.s002.pdf]

## Brain cortex

## Pancreatic tissue

**CB1R**

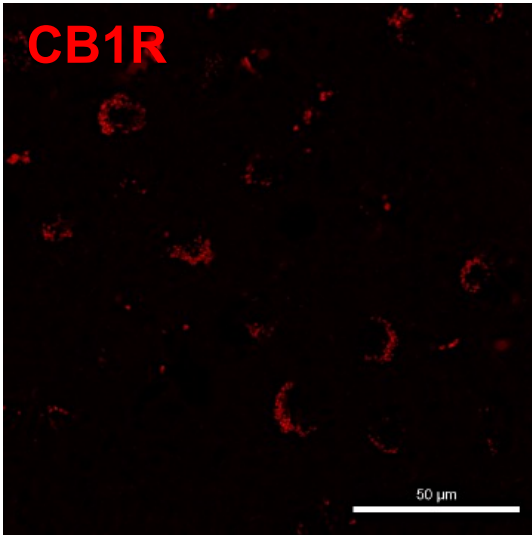

**Isotype control**

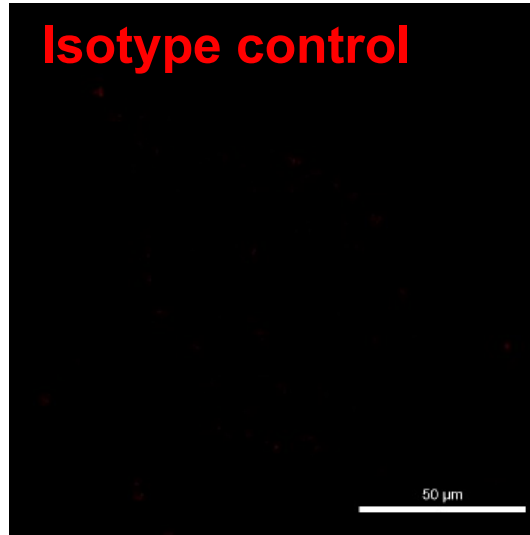

**CB1R**

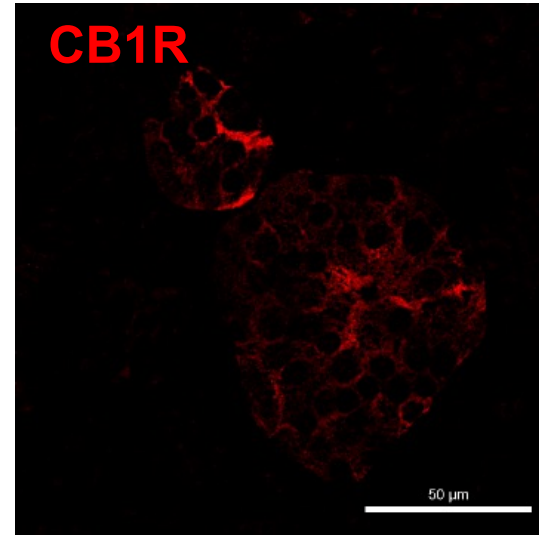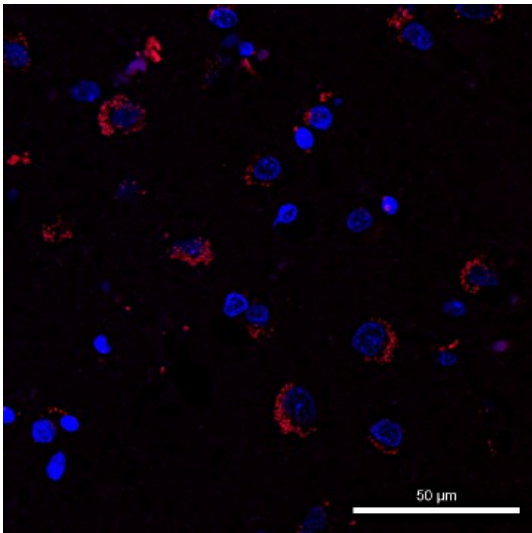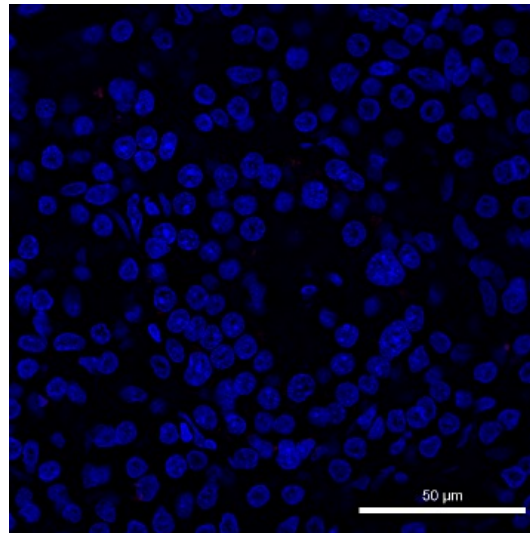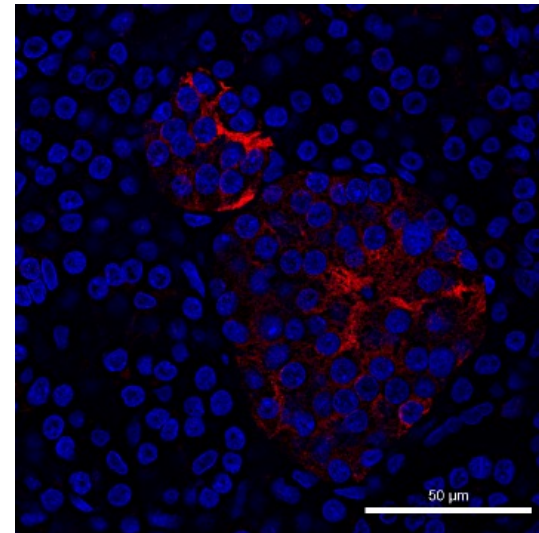

Supplement: S3 Fig — Immunofluorescent staining of human brain cortex tissue and pancreatic tissue from individuals with a history of chronic marijuana use. The sections were stained for CB1R (red). Isotype control was also used. Nuclei are shown in blue. Confocal images were taken using Zeiss LSM 700. Scale bars, 50 μm. (PDF) [file pone.0258434.s003.pdf]
